# Achieving osteoporosis treat-to-target goals with teriparatide or alendronate: sub-analysis of Japanese Osteoporosis Intervention Trial-05 (JOINT-05)

**DOI:** 10.1007/s00774-024-01515-5

**Published:** 2024-05-16

**Authors:** Hiroshi Hagino, Shiro Tanaka, Tatsuhiko Kuroda, Satoshi Mori, Satoshi Soen

**Affiliations:** 1 Department of Rehabilitation, Sanin Rosai Hospital, 1-8-1 Kaikeshinden, Yonago, Tottori 683-8605 Japan; 2 Department of Clinical Biostatistics, Graduate School of Medicine, Kyoto University, Kyoto, Japan; 3 Public Health Research Foundation, Shinjuku-Ku, Tokyo, Japan; 4 Seirei Hamamatsu General Hospital, Hamamatsu, Japan; 5 Soen Orthopaedics, Osteoporosis and Rheumatology Clinic, Kobe, Hyogo Japan

**Keywords:** Treat-to-target, Bone mineral density, Measurement site, Osteoporosis, Teriparatide

## Abstract

**Introduction:**

The purpose of this study was to evaluate whether bone mineral density (BMD) ≥ −2.5 SD could be used as the treat-to-target (T2T) goal when treating osteoporosis with teriparatide (TPTD) and alendronate (ALN), and to investigate the relationship with incident vertebral fracture by re-analyzing data from a randomized, controlled trial (JOINT-05) involving postmenopausal Japanese women at high fracture risk.

**Materials and methods:**

Participants received sequential therapy with once-weekly TPTD for 72 weeks, followed by ALN for 48 weeks (TPTD-ALN group) or ALN monotherapy for 120 weeks (ALN group). BMDs were measured at the lumbar spine (L2-4), total hip, and femoral neck at 0, 24, 48, 72, and 120 weeks by dual-energy X-ray absorptiometry. The T2T goal was BMD ≥ −2.5 SD, and the endpoint was the proportion of participants with baseline BMD < −2.5 SD in three measurement sites achieving BMD ≥ −2.5 SD.

**Results:**

A total of 559 participants were selected. BMD ≥ −2.5 SD at 120 weeks in the L2-4, total hip, and femoral neck sites was achieved in 20.5%, 23.1%, and 5.9%, respectively, in the TPTD-ALN group and 22.2%, 11.7%, and 7.3%, respectively, in the ALN group. Incident vertebral fractures occurred in areas of both lower and high BMD.

**Conclusion:**

During the 1.5-year treatment period, more than 20% of participants achieved BMD ≥ −2.5 SD as a T2T goal at L2-4. Since the achievement level differed depending on the BMD measurement site, the appropriate site should be selected according to the baseline BMD level.

## Introduction

Osteoporosis is a systemic skeletal disorder characterized by low bone mass, deterioration of bone tissue microarchitecture, disruption of bone architecture, an increase in bone fragility, and an increased fracture risk [1]. Fragility fractures impose a substantial burden on societies worldwide [1], particularly hip and clinical vertebral fractures, which can result in significant morbidity (e.g., decreased mobility, pain, reduced quality of life) and increase the risks of mortality, hospitalization, and nursing home use [2–4]. Several pharmacological agents are available to lower fracture risk, either by reducing bone resorption or by stimulating bone formation [5].

Treat-to-target (T2T) is a clinical strategy with a treatment endpoint as the target and is used particularly for long-term treatment. In the T2T approach, if treatment results in failure to achieve the target, the next enhanced treatment protocol is selected [6, 7]. Use of a T2T approach may improve treatment adherence or disease progression by making patients aware of their own goals [8, 9].

T2T efforts are especially important in osteoporosis because the duration of treatment is prolonged. Previously, the target of treatment for osteoporosis was discussed, and candidates for targets have included the Fracture Risk Assessment Tool (FRAX®), bone turnover markers, and osteoporotic fracture [10–12].

Although BMD is also a candidate goal for T2T, no consensus has been reached on its threshold. The diagnosis of osteoporosis uses a BMD T-score of less than −2.5 SD as the threshold, which is calculated from the mean value for the young adult population. Therefore, there are many cases in which participants having lower BMD do not exceed −2.5 SD. It is considered important to know how many participants exceed −2.5 SD by treatment protocol.

BMD has been measured at multiple sites, including the lumbar spine and femur, and a relationship between increased site-specific BMD and fracture risk reduction has been reported [13].

In the present analysis, data from JOINT-05 [14, 15], which was a randomized, controlled trial comparing the efficacies of teriparatide (TPTD) and alendronate (ALN), were used to evaluate: whether BMD > −2.5 SD could be used as the T2T goal for treatment; the difference by BMD measurement site; and the relationship of change in BMD to incident fracture.

## Materials and methods

### Participants and measurements.

JOINT-05 enrolled Japanese women aged 75 years or older with primary osteoporosis [16]. The participants were randomly assigned (1:1) to receive sequential therapy with TPTD for 72 weeks followed by ALN for 48 weeks (TPTD-ALN group) or monotherapy with ALN for 120 weeks (ALN group). TPTD 56.5 μg was injected once-weekly, and ALN was provided as a 5 mg tablet (once daily), 35 mg tablet or jelly (once weekly), or 900 μg infusion (once every 4 weeks). Native vitamin D 400 supplements were provided to both groups throughout the entire treatment period.

In order to evaluate participants who were adequately treated with medication, from the full analysis set of JOINT-05, participants with an adherence rate greater than 70% were selected for this analysis. Adherence to treatment was assessed in categories of: (1) almost 100% adherence; (2) about 50% adherence; (3) less than 33% adherence; and (4) no adherence.

For this analysis, data of BMD (T-score) and the incidence of vertebral fracture were used. BMDs at the lumbar spine (L2-4), total hip, and femoral neck were measured at 0, 24, 48, 72, and 120 weeks by dual-energy X-ray absorptiometry (DXA) in each institution. BMD was not measured for all participants, and measurement sites varied according to the type of DXA. Incidences of vertebral fractures during the observation period were assessed morphologically [17].

It has been previously reported that once BMD exceeded -2.5 SD with osteoporosis drug treatment, the incidence of subsequent fractures was decreased [18]. Therefore, in this analysis, the target was set to BMD ≥ −2.5 SD. The endpoint of this analysis was the proportions of participants with baseline BMD < −2.5 SD at three measurement sites achieving BMD ≥ −2.5 SD as the T2T goal.

### Statistical analysis

Numerical and categorical data are described by means and standard deviation (SD) and proportions, respectively. A waterfall plot was used to visualize the relationship between BMD achievement level and incident vertebral fractures. Within-group change in BMD was examined by the paired *t*-test, and differences between groups in mean BMD and mean change in BMD were examined by the unpaired *t*-test. Proportions of participants with baseline BMD < −2.5 SD in each group achieving BMD ≥ −2.5 SD were compared by Fisher’s exact test. All data were analyzed with SAS software version 9.4 (SAS Institute, Cary, NC). All P values < 0.05 were considered significant.

## Results

A total of 559 participants were selected for this analysis. The baseline characteristics of the TPTD-ALN group (N = 202) and of the ALN group (N = 357) are shown in Table 1. The mean age was 81 years in both groups, approximately 70% of participants had prevalent vertebral fractures, and the percentage with a maximum fracture grade of 3 was approximately 40%. At baseline, the proportions of participants in the TPTD-ALN group and ALN group with: L2-4 BMD < −2.5 SD were 46.3% and 53.0%, respectively; total hip BMD < −2.5 SD were 59.0% and 61.2%, respectively; and femoral neck BMD < −2.5 SD were 77.9% and 76.4%, respectively.

**Table 1:** Baseline characteristics of postmenopausal women with severe osteoporosis

	TPTD-ALN group (N = 202)	ALN group (N = 357)
Age, y	81.2 ± 4.3	81.5 ± 4.7
Age at menopause, y	49.5 ± 4.2	49.3 ± 4.3
BMI, kg/m^2^	22.3 ± 3.7	22.3 ± 3.4
Number of prevalent vertebral fractures	1.8 ± 2.0	1.9 ± 2.1
0	30.2%	30.8%
1	27.7%	24.9%
2	15.8%	15.4%
3	10.4%	10.1%
4	5.4%	6.7%
5 or more	10.4%	12.0%
Maximum grade of prevalent vertebral fractures		
Grade 1	10.4%	9.0%
Grade 2	16.3%	19.0%
Grade 3	43.1%	41.2%
History of hip fractures, yes, %	14.4%	12.9%
L2-4 BMD, T-score	−2.3 ± 1.4	−2.4 ± 1.4
L2-4 BMD, < −2.5 SD, %	46.3%	53.0%
Total hip BMD, T-score	−2.74 ± 0.99	−2.79 ± 1.04
Total hip BMD, < −2.5 SD, %	59.0%	61.2%
Femoral neck BMD, T-score	−3.28 ± 1.01	−3.15 ± 1.06
Femoral neck BMD, < −2.5 SD, %	77.9%	76.4%
Prior treatment for osteoporosis, yes, %	55.4%	58.0%
Prior bisphosphonates, yes, %	29.7%	31.7%

Values are shown as means ± SD or percentages. *BMI* body mass index; *BMD* bone mineral density; *SD* standard deviation; *TPTD* teriparatide; *ALN* alendronate

**Fig. 1:**
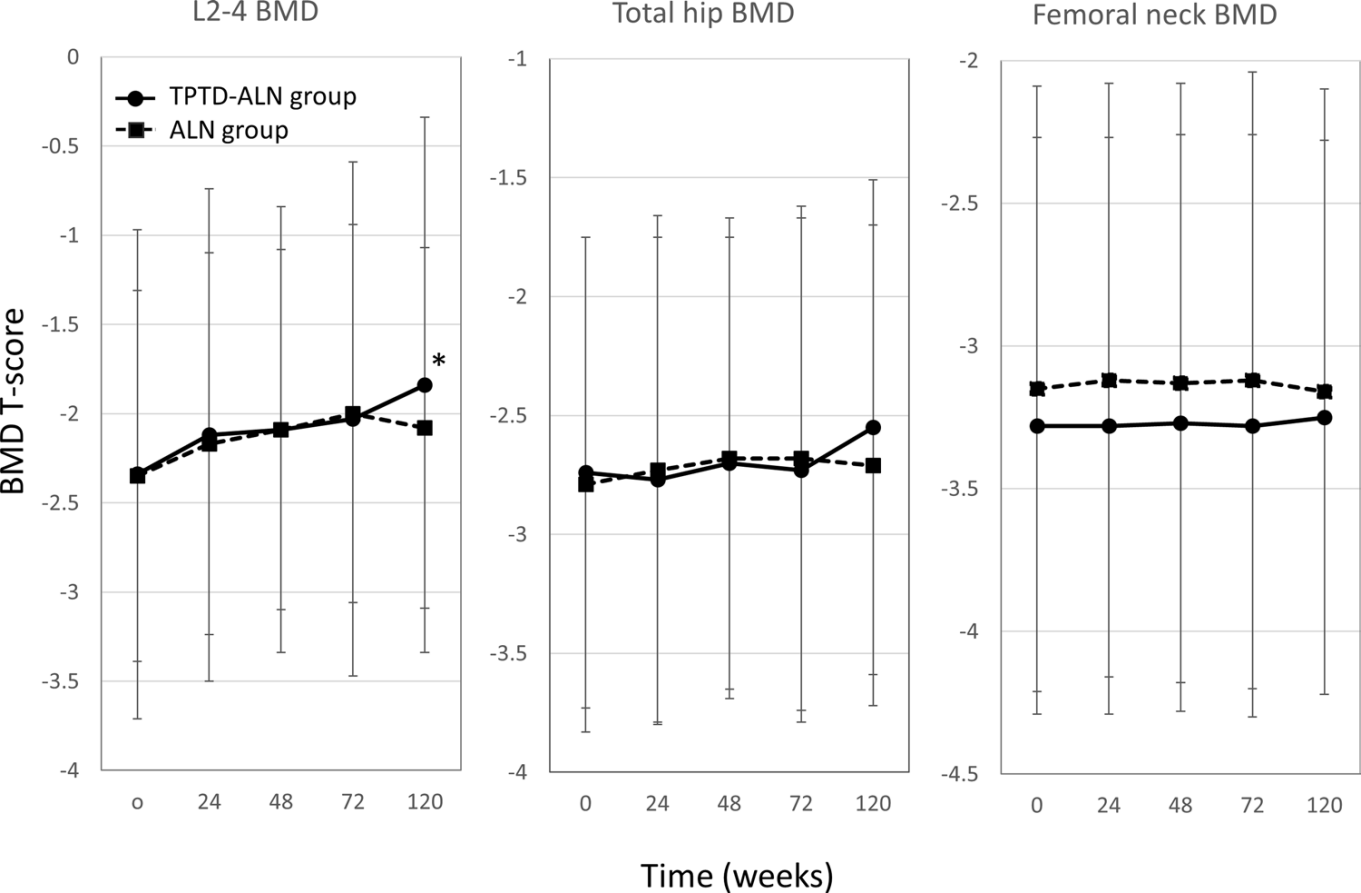
Change in BMD by treatment group and measurement site. Changes in T-score from baseline in L2-4, total hip, femoral neck BMDs are shown in the teriparatide to alendronate (TPTD-ALN) and alendronate (ALN) groups by measurement week. Data are mean percent change ± standard deviation. *P < 0.05 for comparison with 0 weeks

Changes of BMD at the 3 measurement sites are shown in Fig. 1. There were no significant differences in BMD between treatment groups at any time and at each measurement site. L2-4 BMD at 120 weeks increased significantly compared to baseline in the TPTD-ALN group (P = 0.02).

Proportions of participants with baseline BMD < −2.5 SD achieving BMD ≥ −2.5 SD are shown in Table 2. In the TPTD-ALN group, 13.6% of participants at 72 weeks and 20.5% at 120 weeks achieved L2-4 BMD ≥ −2.5 SD. In addition, total hip BMD ≥ −2.5 SD was achieved by 15.2% of the participants at 72 weeks and by 23.1% at 120 weeks, and femoral neck BMD ≥ −2.5 SD was seen in 5.9% at 120 weeks. In the ALN group, L2-4 BMD ≥ −2.5 SD was achieved in 15.6% at 72 weeks and 22.2% at 120 weeks. The overall achievement rates for total hip and femoral neck BMDs ≥ −2.5 SD at 120 weeks were 11.7% and 7.3%, respectively. There was no significant difference in the proportions between treatment groups.

**Table 2:** Proportions of participants with baseline BMD < −2.5 SD who achieved BMD ≥ −2.5 SD

Measurement site	TPTD-ALN group			ALN group			P
	Frequency	Proportion	N	Frequency	Proportion	N	
L2-4 BMD							
24 weeks	5	9.1%	55	14	13.6%	103	0.46
48 weeks	8	14.8%	54	19	18.3%	104	0.66
72 weeks*	6	13.6%	44	12	15.6%	77	1.00
120 weeks*	9	20.5%	44	16	22.2%	72	1.00
Total hip BMD							
24 weeks	6	13.0%	46	3	4.1%	73	0.09
48 weeks	7	15.6%	45	6	8.1%	74	0.24
72 weeks	7	15.2%	46	7	9.6%	73	0.39
120 weeks	9	23.1%	39	7	11.7%	60	0.17
Femoral neck BMD							
24 weeks	4	4.2%	95	7	5.3%	131	0.76
48 weeks	3	3.2%	94	8	6.0%	134	0.53
72 weeks	5	5.3%	95	9	7.1%	126	0.78
120 weeks	5	5.9%	85	8	7.3%	109	0.78

*BMD* bone mineral density; *SD* standard deviation; *TPTD* teriparatide; *ALN* alendronate. *Cases of vertebral fracture were excluded

**Fig. 2:**
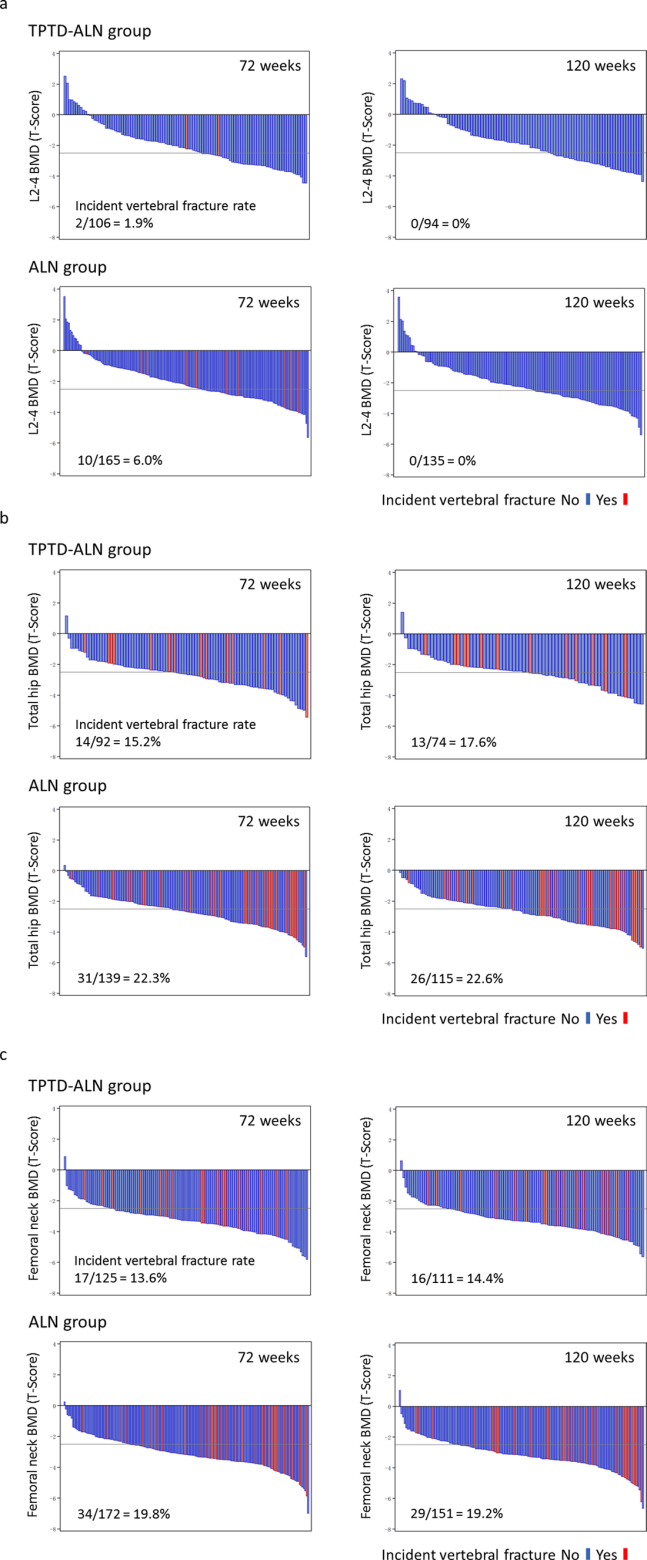
Waterfall plot for the relationship between BMD status and incident vertebral fractures, L2-4 **(a)**, total hip **(b)**, and femoral neck **(c)** BMD T-scores and incident vertebral fractures in the teriparatide to alendronate (TPTD-ALN) group and the alendronate (ALN) group at 72 weeks and 120 weeks

The results of the waterfall plot of the relationship between the BMD distribution and incident fracture are shown in Fig. 2a–c. It was found that incident vertebral fractures occurred in the areas of lower BMD, as well as in areas of high BMD.

## Discussion

In this analysis, the rates of achieving BMD ≥ −2.5 SD as the T2T goal by TPTD and ANL treatment were evaluated. It was found that, in participants with L2-4 BMD < −2.5 SD at baseline, 13.6% achieved the target after 72 weeks of treatment with TPTD, and 20.5% achieved the target with a subsequent switch to ALN at 120 weeks. In the group that received ALN treatment for 120 weeks, 22.2%, 11.7%, and 7.3% of participants achieved the target L2-4, total hip, and femoral neck BMDs, respectively.

The achievement of T2T goals with TPTD has already been reported using the data from the TOWER trial [19]. The proportions achieving the goal BMDs were 21.9% for L2-4 BMD and 14.5% for femoral neck BMD at 72 weeks [19], higher than the current results. The differences may be explained by differences in the participants’ ages. The mean age of the participants was 74 years in the TOWER trial and 81 years in JOINT-05.

Compared by measurement site, the proportion achieving the T2T BMD goal was relatively low at the femoral neck site compared to the L2-4 and total hip sites. One reason for the difference was that the baseline T-score was lower at the femoral neck than at the other sites. If BMD was measured at more than one site, the site more likely to achieve the goal should be selected.

Recently, European experts convened a consensus conference and compiled a report on T2T goals for osteoporosis [20]. There was also consensus that a T2T strategy is applicable to osteoporosis, and that BMD is currently the most clinically appropriate target. With regard to the definition of a specific BMD treatment target and timeframes applicable to T2T, no clear consensus was reached; experts emphasized that these would need to be individually determined.

In the present analysis, about half of the participants had L2-4 BMD < −2.5 SD at baseline (46.3% in the TPTD-ALN group and 53.0% in the ALN group), and 20% of participants achieved BMD ≥ −2.5 SD after 120 weeks of treatment. According to the American Society of Bone and Metabolism and the United States National Osteoporosis Foundation (NOF), patients recommended to be offered drugs, such as bisphosphonate that have at least a 50% chance of achieving their goals within 3 to 5 years of starting treatment [12]. The remaining 80% of participants require further follow-up at least 3 years, and changes in treatment should be considered based on periodic BMD measurements. Moreover, another target should be set for participants with L2-4 BMD ≥ −2.5 SD at baseline.

In this analysis, the relationship between the BMD level and incident vertebral fractures was also confirmed. With TPTD treatment, the incidence of fractures was not necessarily dependent on BMD status. It may be due to the change of bone quality other than BMD. In fact, TPTD has been reported to increased material properties of bone [21]. Factors of bone quality other than changes in BMD that affect bone strength may also need to be taken into account in treatment.

This study had several limitations. The current study included data of participants in the JOINT-05 study, which included patients with osteoporosis and a high fracture risk. Because ALN is also used in osteoporotic patients with low fracture risk, the T2T goal achievement rate with ALN in particular may need to be re-evaluated in patients with a wider range of fracture risk. Not all 3 BMD sites were measured in all participants, and the number of participants for whom BMD was measured varied. The achievement rate in total hip BMD was higher in the TPTD-ALN group (23.1%) than in the ALN group (11.7%), but it was not statistically significant. Although this difference may be due to differences in therapeutic agents, it should be verified by studies with appropriate sample sizes. The background factors contributing to the difference between T2T and non-T2T achievers were unclear. More detailed studies may be needed that include information on patients' bone metabolic turnover and lifestyle habits. Incident vertebral fractures were evaluated in all participants, but BMD was not measured in all of them. Therefore, the relationship between BMD status and fracture incidence was not robust. However, the trends in incident fracture at the 3 measurement sites were identical, suggesting the generalizability of the results.

In conclusion, during the 1.5-year treatment period, more than 20% of participants with osteoporosis achieved BMD ≥ −2.5 SD as a T2T goal. Since the achievement level differed depending on the site of BMD measurement, the appropriate site should be selected according to the baseline BMD level of the patient.

## Data Availability

The data that support the findings of this study are available from the corresponding author upon reasonable request.
